# Hemodynamic disclosure of septic shock patients by intensive care ultrasound

**DOI:** 10.1186/cc12124

**Published:** 2013-03-19

**Authors:** P Theerawit, Y Sutherasan, T Hongpanat

**Affiliations:** 1 Ramathibodi Hospital, Mahidol University, Bangkok, Thailand

## Introduction

Even though invasive hemodynamic devices are usually used for assessment of septic shock victims, they cannot evaluate the heart function. LV dysfunction as well as right heart syndrome are not uncommon in sepsis and critical patients. Intensive care ultrasound discloses these data and leads to appropriate treatment.

## Methods

The study was a prospective cross-sectional study. The measurement was performed within 24 hours of ICU admission. We excluded patients with history of COPD and pulmonary hypertension from any diseases. Only good-quality images acquired from subjects were included for analysis. The primary objective was to disclose how the hemodynamic changed in septic patients by ICU-US.

## Results

A total of 133 septic patients were measured by ICU-US. Good image quality was acquired in 115 cases (86.47%). The mean ages were 57.48 ± 17.87 years. The three major causes of sepsis were pneumonia, unknown source, abdominal infection and bacteremia. Heart failure at admission was found only in 1.79%. Previous history of hypertension, DM, and coronary artery disease was found in 12.17%, 11.30%, and 1.74% of patients. The mean LV ejection fraction (LVEF) was 54 ± 15.43%. The percentages of patients with LVEF <35%, 35 to 40%, 41 to 45% and >45% were 13.2%, 7.5%, 8.5%, and 70.8% respectively. Diastolic dysfunction defined by E/A ratio <1 was observed in 47.5% of patients. A total 44.6% of cases had cardiac output under 4 l/minute whereas CO over 6 l/minute was found in 18.1% of cases. The average mean pulmonary artery pressure was 34.75 ± 15.13 mmHg. The proportion of patients with meanPAP over 25 mmHg was 76.2%. RV to LV ratio >1 was found in 42.4% of septic patients.

## Conclusion

Cardiac dysfunction, namely left ventricle and probably right ventricle, was not uncommon in septic shock patients. Without intensive care ultrasound, all crucial information was delayed until patient deterioration and initial treatment may be harmful. Thus cardiac ultrasound should be used initially to disclose hemodynamic features before routine resuscitation is initiated.
